# Appraisal of the DIERS method for calculating postural measurements: an observational study

**DOI:** 10.1186/s13013-017-0134-y

**Published:** 2017-09-26

**Authors:** Brian Degenhardt, Zane Starks, Shalini Bhatia, Gwyn Kelley-Franklin

**Affiliations:** 0000 0004 0383 094X grid.251612.3 A.T. Still University, 800 W. Jefferson St, Kirksville, 63501 Missouri USA

**Keywords:** DIERS formetric 4D, Postural sway, Posture, Rasterstereography, Spine shape, Surface topography

## Abstract

**Background:**

Surface topography is increasingly used with postural analysis. One system, DIERS formetric 4D, measures 40 defined spine shape parameters from a 6-s scan. Through system algorithms, a set of spine shape parameter values from 1 of 12 recorded images obtained during a scan becomes the DIERS-reported value (DRV) for postural assessment. The purpose of the current study was to compare DRV with a standard average value (SAV) calculated from all 12 images to determine which method is more appropriate for assessing postural change.

**Methods:**

One mannequin and 30 human participants were scanned over 5 days. Values from each image and the DRV for 40 defined spine shape parameters were exported, and mean DRV, mean SAV, mean DRV, and within-scan variance were calculated. Absolute difference and percent change between mean DRV and mean SAV were calculated for the mannequin and humans. Inter-method reliability was calculated for humans. Within-scan variance for each parameter was tested for significant variability.

**Results:**

For all spine shape parameters on the mannequin, absolute difference (< 0.6 mm, 0.1°, or 0.1%) and percent change (< 2.90%) between mean DRV and mean SAV for each parameter were small. Nine parameters on human participants had a large percent change (> 7%). Absolute difference between mean DRV and mean SAV for those nine parameters was small (≤ 0.87 mm or 0.61°). Absolute difference for all other parameters ranged from 0.02 to 6.98 mm for distance measurements, from 0.01 to 1.21° for angle measurements, and from 0.15 to 0.22% for percentage measurements. Inter-method reliability between DRV and SAV was excellent (0.94–1.00). For the mannequin, within-scan variance was small (< 1.62) for all parameters. For humans, within-scan variance ranged from 0.05 to 36.04 and was different from zero for all parameters (all *P* < 0.001).

**Conclusions:**

The minimal variability observed in the mannequin suggested the DIERS formetric 4D instrument had high within-scan reliability. The DRV and SAV provided comparable spine shape parameter values. Because within-scan variability is not reported with the DRV, the clinical usefulness of current DRV values is limited. Establishing an estimate of variance with the SAV will allow clinicians to better identify a clinically meaningful change.

## Background

Surface topography has recently gained popularity for the assessment of postural deformities. One method of surface topography, called rasterstereography, was developed by Drerup and Hierholzer in the 1980s [1]. This radiation-free technique projects horizontal stripes of light onto the surface of the participant’s back, and static images of the lines are recorded and digitized. Based on the distortion of the projected horizontal lines, a three-dimensional image of the surface of the back can be produced, measured, and correlated with underlying spinal curve deformities [2–5]. Because of its non-invasive, non-contact, and radiation-free ability to observe posture and spinal deformities, one surface topography instrument, the DIERS formetric 4D (DIERS Medical Systems, Chicago, IL), allows researchers to observe a full profile of posture changes without the hazards associated with radiography. As such, it has increasingly been used in clinical practice.

Using the DIERS formetric 4D, a typical scan of the back for static standing posture analysis takes 6 s. During a scan, 12 images are collected of the posterior trunk. With each scan, the surface topography instrument calculates 40 defined shape parameters based on angles, distances, rotations, and deviations of the spine and pelvis. To determine the individual shape parameters reported from the series of images, an algorithm calculates average values from the entire scan for specific parameters. As a means of data reduction, the algorithm selects 1 of the 12 images closest to the average values and reports the spine shape parameter values for that image.

In general, there are two potential factors that contribute to variability in any instrument’s output: the equipment and the patient being observed. For the DIERS formetric 4D, postural sway and body movement caused by respiration are sources of variability and are observed even when a person is standing still. While data reduction is a common practice with big datasets, it is unclear whether the above data reduction process, which prioritizes presenting data consistent with a graphical image, creates limitations when using the instrument longitudinally in the clinical and research arenas. By calculating the reported spine shape parameters from only one image, the data from each of the remaining images are ignored. Without this additional data, it is impossible to assess the precision or variability within a scan. Further, this data may influence the degree of change that is needed to identify real postural change when comparing studies over time. Studies involving patients with adolescent idiopathic scoliosis [2, 6] and without spinal deformities [7–10] have reported the reliability of the DIERS formetric 4D, but it is unclear whether reported values were the DIERS-reported value (DRV) from a single image or whether the values from each image were exported and averaged to a standard average value (SAV) for each of the spine shape parameters.

The purpose of the current study was to compare the algorithm-selected DRV with the SAV calculated from the 12 images recorded during a single scan to determine which method is more appropriate for evaluating data. To our knowledge, no other study has evaluated a standard averaging method for the defined spine shape parameters available to the clinician. Rather than presenting the results from the image that most closely represents the average values of the images, we believe a true average of all values with an assessment of variability will be more clinically meaningful. As such, we hypothesized that, when scanning a human-shaped mannequin, minimal variability would be inherent within the instrument. Further, variability from postural sway and respiration in human participants would affect the spine shape parameter values. Therefore, representing the spine shape parameters as SAV with an indication of within-scan variability would be a more effective method for observation of postural changes in longitudinal and interventional studies.

## Methods

For the current observational study, 30 male and female participants aged 18 to 65 years were recruited through campus email, posters, and word-of-mouth. Potential participants were excluded if they had a history of surgery to the spine or back tattoos, were unable to stand without assistance, or had a body mass index (BMI) above 35 or below 20. All participants reported to a university research laboratory and completed an approved informed consent form before participating. The local institutional review board approved all aspects of the study.

**Fig. 1:**
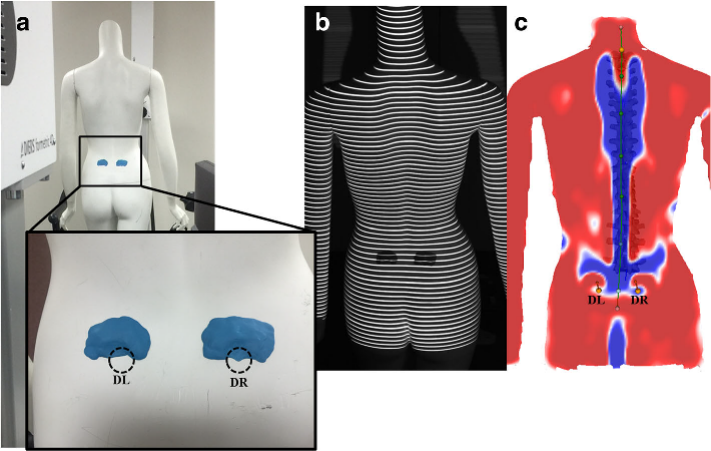
Example of DIERS formetric 4D surface topography scan using a mannequin. **a** The left (DL) and right sacral dimples (DR) associated with the posterior superior iliac spine were added to the mannequin using modeling clay before scanning (*inset*). **b** A grid of lines is projected onto the surface of the back and images are captured by the DIERS formetric 4D instrument. **c** The technician verified that DL and DR were clearly and accurately localized on the 3D model created by the DIERS formetric 4D instrument

As a control, an adult-sized female mannequin was scanned daily for 7 days. The mannequin eliminated the human factor of postural sway and breathing and allowed for evaluation of the variability inherent to DIERS formetric 4D instrument. Any observed variability in those scans would indicate variability from the instrument rather than the more variable human form. To more appropriately approximate the human form, the two sacral dimples near the posterior superior anterior spines were modified on the mannequin using modeling clay (Fig. 1).

Before being scanned, human participants completed a short medical history and a demographic questionnaire. They then removed all clothing except for a pair of shorts so that the entire surface of the back was exposed from the top of the gluteal cleft to the base of the hairline. Participants were positioned on a platform 2 m from the DIERS formetric 4D projection unit. The heels of their bare feet were placed on the platform, so they were touching a plastic tube reference line. The plastic tube was secured perpendicular to the surface of the platform to ensure consistent anterior and posterior foot placement at a standardized distance from the camera, but it did not inhibit any natural hip internal or external rotation. After foot placement, the participants were asked to stand in a relaxed, natural position. In front of the participants, an adjustable fixed point was provided as a visual reference and was based on the shoulder height of the participants. Participants were instructed to focus their gaze on this fixed point during the scans to control head position. Thirty scans were completed for each participant over 5 days. Participants were scanned six times consecutively before moving from the platform. During each 6-s scan, participants were asked to stand naturally. Between scans, participants were asked not to move from the original position on the platform. The time to complete six scans was less than 6 min.

Each scan was completed in the DIERS data collection and processing software, DiCAM III, in the 4D average module. During each scan, 12 images were recorded over the 6 s (2 Hz). For each image, up to 50,000 points were captured, digitized, and analyzed automatically by the DIERS formetric 4D instrument. From each of the images, 40 spine shape parameters were exported for evaluation. These parameters were sorted into five subgroups based on the clinical relatedness of the parameter. These subgroup parameters included localization and distance, trunk and pelvis imbalances, spinal reference points, spinal curve measurements, and spinal deviation (Table 1). Spine shape parameters are reported in millimeters, percentage, or degrees depending on the specific parameter.

**Table 1:** Spine shape parameters output by the DIERS formetric 4D instrument and their definitions

Spine shape parameters by subgroup	Definition
Localization and distance	
Trunk length VP-DM, mm	The distance from VP to DM
Trunk length VP-SP, mm	The distance from VP to the SP
Trunk length VP-SP, %	The distance of VP-SP expressed as a percentage of VP-DM
Dimple distance, mm	The distance from DL to DR
Dimple distance, %	The distance of DL to DR expressed as a percentage of VP-DM
Trunk and pelvis imbalances	
Trunk inclination VP-DM, °	The angle between the line connecting VP-DM and an external vertical line
Trunk inclination VP-DM, mm	The distance between VP and the connecting external vertical line
Trunk imbalance VP-DM, °	The angle between the line connecting VP-DM and a vertical line through VP
Trunk imbalance VP-DM, mm	The lateral distance between VP and DM
Pelvic tilt DL-DR, °	The angle between the line connecting DL and DR and an external horizontal line
Pelvic tilt DL-DR, mm	The difference in height between DL and DR
Pelvic torsion DL-DR, °	The torsion of the surface normals of DL and DR
Pelvic inclination dimples, °	The mean vertical components of the surface normals at DL and DR
Rotation correction pelvis, °	In the frontal plane the angle of rotation of DR in the frontal plane in relation to DL
Spinal reference points	
Inflection point ICT, mm	The point of maximum positive surface inclination above the KA
Kyphotic apex, mm	The location of the posterior apex of the sagittal profile
Inflection point ITL, mm	The point of maximum negative surface inclination between the KA and the LA
Lordotic apex, mm	The location of the frontal apex of the sagittal profile in the lower region
Inflection point ILS, mm	The point of maximum positive surface inclination in the region between the LA and the sacrum
Fleche cervicale, mm	The horizontal distance between the cervical apex and the tangent through the KA
Fleche lombaire, mm	The horizontal distance between the LA and the tangent through the KA
Fleche cervicale VP, mm	The horizontal distance between the VP and the KA
Spinal curve measurements	
Kyphotic angle ICT-ITL, °	The angle between the surface tangents from the ICT and ITL
Kyphotic angle VP-ITL, °	The angle between the surface tangents from VP and ITL
Kyphotic angle VP-T12, °	The angle between the surface tangents on VP and the location of the calculated T12
Lordotic angle ITL-ILS, °	The angle between the surface tangents from ITL and ILS
Lordotic angle ITL-DM, °	The angle between the surface tangents from ITL and DM
Lordotic angle T12-DM, °	The angle between the surface tangents from T12 and DM
Pelvic inclination, °	The angle of the vertical surface normals from the horizontal of DM
Spinal deviation	
Surface rotation RMS, °	The RMS of the horizontal components of the surface normals on the symmetry line
Surface rotation, °	The maximum value of the horizontal components of the surface normals on the symmetry line
Surface rotation right °	The maximum value of the horizontal components of the surface normals on the symmetry line to the right
Surface rotation left, °	The maximum value of the horizontal components of the surface normals on the symmetry line to the left
Surface rotation amplitude, °	The maximal spinal torsion calculated from the maximal rotation to the right and the left
Trunk torsion, °	The maximal value of the horizontal components on VP compared to the horizontal components of the symmetry line on DM
Lateral deviation RMS, mm	The RMS deviation of the midline of the spine from the direct connection of VP-DM in the frontal plane
Lateral deviation, mm	The maximum deviation of the midline of the spine from the direct connection of VP-DM in the frontal plane
Lateral deviation right, mm	The maximum deviation of the midline of the spine from the VP-DM line to the right
Lateral deviation left, mm	The maximum deviation of the midline of the spine from the VP-DM line to the left
Lateral deviation amplitude, mm	The sum of the maximum deviation of the right and the left lateral deviation values

Spine shape parameter definitions adapted from DIERS formetric III 4D Manual (Created 21.06.2010, Revision grade 5) and DIERS Optical Measurement of the Spine Information for the Assessment (Version 1, Created 04.08.2009)

*DL* left sacral dimple, *DM* middle point between the left and right sacral dimples, *DR* right sacral dimple, *ICT* cervical-thoracic inflection point, *ILS* lumbar-sacral inflection point, *ITL* thoracic-lumbar inflection point, *KA* kyphotic apex, *LA* lordotic apex, *RMS* root mean square, *SP* sacral point, *VP* vertebral prominens

**Fig. 2:**
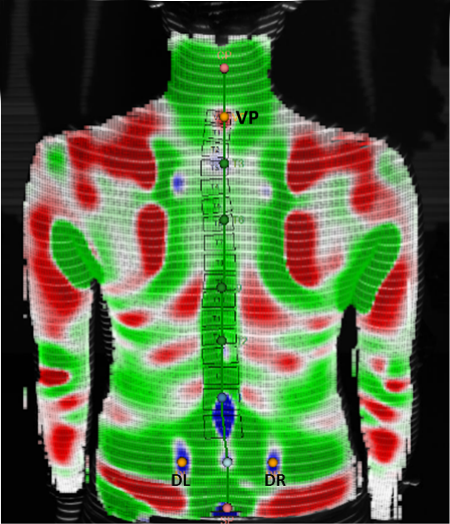
Example of correct landmark localization by DIERS formetric 4D scan on a human participant. The technician verified that the automatically localized points of the left sacral dimple (DL) and right sacral dimple (DR) were within the concave dimples of the lower back (*blue*), and the vertebral prominens (VP) was within the convex region just below the neck (*red*) on each image from each scan

Each scan was processed as per the manufacturer’s instructions. On each of the collected images, the software automatically indicated the location of the left (DL) and right (DR) sacral dimples associated with the posterior superior iliac spine [1] and the location of the vertebral prominens (VP), which is typically located at C7 [11]. The middle point between the dimples (DM) was determined from the location of DL and DR. Since accurate localization of these reference landmarks is vital for accurate spinal reconstruction, the location of these points was confirmed by the technician. The positions of DL and DR were represented by round, blue areas and indicated by a concave dimple on both sides of the spine near the posterior superior iliac spine (Fig. 2). For some scans, the DIERS formetric 4D improperly localized DL and DR markers on the participant’s shorts or within concave areas outside of the actual dimple area. This problem was addressed by cropping at the lower edge of the image. The system then reprocessed the image and relocated the landmarks. If this method still failed to locate the dimples, the markers representing those dimples were manually moved within the DIERS processing program to their proper relative position, and the image was reprocessed so the marker was at the deepest point of the dimple. The VP was represented by a convex region at the base of the neck (Fig. 2). In a similar fashion, if the VP marker fell outside of this area, it was moved to the proper position at the most prominent portion of the convex region. Although the cropping tool is also available at the top of the image, cropping was not used to relocate VP. Once it was confirmed by the technician that the points representing DL, DR, and VP were localized within the concave area of the dimples and on the convex area at the base of the neck on each image, no further changes were made to the scan.

To evaluate the SAV, a nested random effects model was built in SAS version 9.4 (SAS Institute, Inc., Cary, NC) for the 40 spine shape parameters (Table 1) using data from the mannequin that was collected on all 5 days and included the six scans from each day and the 12 images produced during each scan. Mean SAV and within-scan variance for each spine shape parameter were calculated from this model. Another nested random effects model was built for the mannequin using only the DRV for each scan rather than the values from each of the 12 images. Mean DRV for each parameter was calculated from this model. Percent change in mean was calculated between the mean SAV and the mean DRV for each parameter using the following formula: $\mid \frac{\mathrm{mean}\  \mathrm{of}\ \mathrm{SAV}-\mathrm{mean}\  \mathrm{of}\ \mathrm{DRV}}{\mathrm{mean}\  \mathrm{of}\ \mathrm{SAV}}\mid \times 100$. Percent change was represented as an absolute value, so values of percent change are all positive.

Similar nested models were built using pooled data from the 30 human participants. Like the mannequin, two different models were built for human participants: one that contained the SAV and one that contained the DRV from each scan. Mean SAV for each parameter, mean SAV for each participant, and within-scan variance were calculated from the first model, and mean DRV for each parameter and mean DRV for each participant were calculated from the second model. Mixed effects analysis of variance models were built using mean SAV and mean DRV for each participant, treating method (SAV or DRV) as a fixed effect, and inter-method reliability was calculated for each of the 40 spine shape parameters using intraclass correlation coefficients (ICC). Percent change for human participants was also calculated between the mean SAV and the mean DRV for each spine shape parameter with the same formula described above. Using data from the human participants, we tested whether the within-scan variance from the 12 images within a scan was significantly different from zero. A significant variance would indicate that respiration and postural sway affected measurements within a scan, so evaluating parameters based on SAV would be more appropriate than using DRV. A *P* value less than 0.05 was considered statistically significant.

## Results

The mannequin was scanned 42 times over 7 days. After the first 2 days, the landmarks were adjusted to ensure that the location of DL and DR were clearly defined. Thirty human participants (age 30.2 ± 9.8 years, BMI 27.3 ± 4.4) completed the current study: 15 males (age 31.9 ± 11.5 years, BMI 27.5 ± 4.1) and 15 females (age 28.6 ± 7.3 years, BMI 27.1 ± 4.8). Each participant completed 30 scans in 5 days for a total of 900 processed and analyzed scans. The location of at least 1 of the landmarks had to be manually adjusted for at least 1 image for 399 (43.33%) of the scans (Table 2).

**Table 2:** Number of scans with landmarks adjusted

Scans	Landmarks adjusted, no. (%)
Total (*n* = 900)	399 (44.33)
Male (*n* = 450)	201 (44.67)
Female (*n* = 450)	198 (44.00)

On the mannequin, the mean DRV and mean SAV for each of the 40 spine shape parameters were similar (Table 3). The absolute difference between the mean DRV and mean SAV for each parameter ranged from 0.01 to 0.55 mm for all distance parameters and from 0.00 to 0.08° for all angle parameters. The percent change ranged from 0 to 2.90%.

**Table 3:** Comparison of means for DIERS-reported values (DRV) and standard average values (SAV) for each parameter

Spine shape parameter by subgroup	Mannequin				Human participants			
	Mean DRV	Mean SAV	Absolute difference	Change (%)	Mean DRV	Mean SAV	Absolute difference	Change (%)
Localization and distance								
Trunk length VP-DM, mm	426.95 (1.23)	426.98 (1.09)	0.03	0.01	463.35 (33.38)	465.83 (33.06)	2.48	0.53
Trunk length VP-SP, mm	462.34 (4.92)	462.47 (2.28)	0.13	0.03	511.31 (35.03)	514.68 (33.69)	3.37	0.65
Trunk length VP-SP, %	108.30 (1.12)	108.32 (1.11)	0.02	0.02	110.34 (1.81)	110.49 (1.77)	0.15	0.14
Dimple distance, mm	74.80 (2.94)	74.88 (1.58)	0.08	0.10	95.64 (11.95)	97.03 (11.06)	1.40	1.44
Dimple distance, %	17.47 (0.69)	17.49 (0.77)	0.02	0.12	20.68 (2.99)	20.90 (2.98)	0.22	1.03
Trunk and pelvis imbalances								
Trunk inclination VP-DM, °	2.41 (0.36)	2.42 (0.64)	0.01	0.37	3.09 (2.25)	3.14 (2.31)	0.05	1.55
Trunk inclination VP-DM, mm	18.26 (2.68)	18.32 (1.74)	0.06	0.33	25.49 (18.32)	25.99 (18.72)	0.50	1.92
Trunk imbalance VP-DM, °	0.78 (1.21)	0.78 (1.20)	0.00	0.62	0.16 (0.85)	0.11 (0.83)	0.04	37.28
Trunk imbalance VP-DM, mm	5.94 (9.16)	5.99 (3.29)	0.05	0.75	1.32 (7.16)	1.00 (7.15)	0.32	31.49
Pelvic tilt DL-DR, °	−2.25 (2.21)	−2.26 (1.67)	0.01	0.50	−0.11 (3.39)	0.02 (3.46)	0.13	719.76
Pelvic tilt DL-DR, mm	−2.98 (2.90)	−3.00 (1.91)	0.02	0.61	0.00 (5.78)	0.17 (5.91)	0.17	100.61
Pelvic torsion DL-DR, °	4.93 (0.71)	4.93 (0.89)	0.00	0.01	0.16 (2.70)	−0.17 (2.51)	0.33	195.92
Pelvic inclination dimples, °	36.47 (1.02)	36.40 (0.91)	0.07	0.18	19.06 (7.38)	17.88 (5.97)	1.18	6.59
Rotation correction pelvis, °	6.18 (1.47)	6.21 (0.91)	0.03	0.47	0.07 (3.14)	−0.32 (2.75)	0.40	123.16
Spinal reference points								
Inflection point ICT, mm	17.95 (2.25)	17.92 (1.68)	0.03	0.16	5.13 (10.40)	4.27 (10.15)	0.87	20.32
Kyphotic apex, mm	−81.19 (21.17)	−80.64 (4.64)	0.55	0.68	−183.81 (36.58)	−190.79 (24.53)	6.98	3.66
Inflection point ITL, mm	−257.38 (2.20)	−257.29 (1.46)	0.09	0.03	−307.74 (36.01)	−311.38 (34.68)	3.64	1.17
Lordotic apex, mm	−326.82 (4.25)	−326.74 (2.23)	0.08	0.02	−384.56 (35.82)	−388.31 (33.38)	3.75	0.97
Inflection point ILS, mm	−420.35 (3.59)	−420.23 (2.13)	0.12	0.03	−460.46 (42.03)	−463.06 (42.21)	2.60	0.56
Fleche cervicale, mm	33.11 (1.58)	33.09 (1.28)	0.02	0.06	71.08 (19.67)	73.69 (17.61)	2.61	3.54
Fleche lombaire, mm	27.14 (1.77)	27.16 (1.40)	0.02	0.06	36.62 (12.62)	37.24 (12.73)	0.62	1.67
Fleche cervicale VP, mm	9.25 (0.93)	9.31 (0.99)	0.07	0.70	45.32 (16.71)	47.71 (14.29)	2.40	5.03
Spinal curve measurements								
Kyphotic angle ICT-ITL, °	35.13 (0.72)	35.13 (0.84)	0.00	0.00	47.23 (9.35)	48.10 (9.05)	0.87	1.81
Kyphotic angle VP-ITL, °	31.53 (0.78)	31.55 (0.87)	0.02	0.08	45.33 (8.95)	46.27 (8.61)	0.94	2.03
Kyphotic angle VP-T12, °	31.47 (0.76)	31.50 (0.88)	0.03	0.08	41.87 (8.44)	42.54 (8.44)	0.68	1.59
Lordotic angle ITL-ILS, °	46.11 (0.63)	46.15 (0.78)	0.04	0.09	36.26 (8.53)	35.59 (8.35)	0.67	1.88
Lordotic angle ITL-DM, °	45.27 (1.08)	45.29 (1.05)	0.03	0.06	34.32 (8.77)	33.57 (8.53)	0.75	2.24
Lordotic angle T12-DM, °	45.24 (1.10)	45.26 (1.06)	0.02	0.05	30.86 (9.27)	29.84 (8.74)	1.02	3.41
Pelvic inclination, °	39.36 (0.50)	39.34 (0.68)	0.03	0.07	21.05 (8.74)	19.84 (7.49)	1.21	6.11
Spinal deviation								
Surface rotation RMS, °	4.56 (0.24)	4.56 (0.44)	0.00	0.02	3.74 (1.24)	3.75 (1.37)	0.01	0.23
Surface rotation, °	11.26 (0.49)	11.28 (0.70)	0.02	0.15	2.35 (7.23)	1.79 (7.20)	0.57	31.68
Surface rotation right, °	11.26 (0.49)	11.28 (0.70)	0.02	0.15	5.97 (3.51)	5.61 (3.43)	0.36	6.41
Surface rotation left, °	−2.20 (0.80)	−2.23 (0.89)	0.04	1.78	−4.38 (2.71)	−4.55 (2.93)	0.18	3.85
Surface rotation amplitude, °	13.49 (0.80)	13.57 (0.99)	0.08	0.60	10.38 (3.05)	10.20 (3.04)	0.18	1.78
Trunk torsion, °	12.08 (0.96)	12.06 (1.41)	0.02	0.18	3.96 (4.35)	3.35 (3.89)	0.61	18.11
Lateral deviation RMS, mm	4.51 (2.11)	4.50 (1.10)	0.01	0.23	5.53 (2.92)	5.59 (2.97)	0.06	0.99
Lateral deviation, mm	7.39 (1.33)	7.36 (1.36)	0.02	0.33	3.87 (10.05)	3.63 (10.28)	0.25	6.79
Lateral deviation right, mm	7.39 (2.08)	7.36 (1.36)	0.02	0.33	7.86 (5.60)	7.88 (5.76)	0.02	0.25
Lateral deviation left, mm	−1.16 (0.43)	−1.19 (0.57)	0.03	2.90	−4.73 (4.11)	−5.01 (4.13)	0.27	5.45
Lateral deviation amplitude, mm	8.59 (1.81)	8.60 (1.27)	0.01	0.15	12.64 (5.34)	12.93 (5.43)	0.29	2.27

DRV and SAV are reported as mean (SD)

*DL* left sacral dimple, *DM* middle point between the left and right sacral dimples, *DR* right sacral dimple, *ICT* cervical-thoracic inflection point, *ILS* lumbar-sacral inflection point, *ITL* thoracic-lumbar inflection point, *RMS* root mean square, *SP* sacral point, *VP* vertebral prominens

For human participants, inter-method reliability between mean DRV and mean SAV was excellent for each of the 40 spine shape parameters (ICC = 0.94–1.00) (Table 4). The absolute difference between the mean DRV and mean SAV for localization and distance subgroup parameters ranged from 1.40 to 3.37 mm for distance parameters and from 0.15 to 0.22% for percentage parameters; the percent change ranged from 0.14 to 1.44% (Table 3). The absolute difference in the trunk and pelvis imbalances subgroup parameters ranged from 0.17 to 0.50 mm for distance parameters and from 0.04 to 1.18° for angle parameters; the percent change ranged from 1.55 to 719.76%. The absolute difference for the spinal reference points subgroup parameters ranged from 0.62 to 6.98 mm, and the percent change ranged from 0.56 to 20.32%. The absolute difference for spinal curve measurements subgroup parameters ranged from 0.67 to 1.21°, and the percent change ranged from 1.81 to 6.11%. The absolute difference for spinal deviation subgroup parameters ranged from 0.02 to 0.29 mm for distance parameters and from 0.01 to 0.61° for angle parameters; the percent change ranged from 0.23 to 31.68%.

**Table 4:** Inter-method reliability for human participants between DIERS-reported value (DRV) and standard average value (SAV)

Spine shape parameter by subgroup	ICC
Localization and distance	
Trunk length VP-DM, mm	0.94
Trunk length VP-SP, mm	0.94
Trunk length VP-SP, %	0.99
Dimple distance, mm	0.99
Dimple distance, %	1.00
Trunk and pelvis imbalances	
Trunk inclination VP-DM, °	1.00
Trunk inclination VP-DM, mm	0.99
Trunk imbalance VP-DM, °	1.00
Trunk imbalance VP-DM, mm	1.00
Pelvic tilt DL-DR, °	0.99
Pelvic tilt DL-DR, mm	0.99
Pelvic torsion DL-DR, °	1.00
Pelvic inclination dimples, °	1.00
Rotation correction pelvis, °	0.99
Spinal reference points	
Inflection point ICT, mm	0.99
Kyphotic apex, mm	0.98
Inflection point ITL, mm	0.97
Lordotic apex, mm	0.97
Inflection point ILS, mm	0.95
Fleche cervicale, mm	1.00
Fleche lombaire, mm	0.98
Fleche cervicale VP, mm	0.99
Spinal curve measurements	
Kyphotic angle ICT-ITL, °	0.99
Kyphotic angle VP-ITL, °	0.96
Kyphotic angle VP-T12, °	0.97
Lordotic angle ITL-ILS, °	0.98
Lordotic angle ITL-DM, °	0.99
Lordotic angle T12-DM, °	0.99
Pelvic inclination, °	1.00
Spinal deviation	
Surface rotation RMS, °	1.00
Surface rotation max, °	0.98
Surface rotation right, °	0.97
Surface rotation left, °	1.00
Surface rotation amplitude, °	0.97
Trunk torsion, °	0.98
Lateral deviation RMS, mm	1.00
Lateral deviation, mm	0.96
Lateral deviation right, mm	0.98
Lateral deviation left, mm	0.95
Lateral deviation amplitude, mm	1.00

*DL* left sacral dimple, *DM* middle point between the left and right sacral dimples, *DR* right sacral dimple, *ICC* intraclass correlation coefficient, *ICT* cervical-thoracic inflection point, *ILS* lumbar-sacral inflection point, *ITL* thoracic-lumbar inflection point, *RMS* root mean square, *SP* sacral point, *VP* vertebral prominens

For the mannequin, the largest absolute difference observed between the mean DRV and mean SAV was for kyphotic apex (0.55 mm) followed by trunk length from VP to the sacral point (0.13 mm) (Table 3). For human participants, the largest absolute difference observed between the mean DRV and mean SAV was for kyphotic apex (6.98 mm) followed by lordotic apex (3.75 mm), inflection point between the kyphotic apex and lordotic apex (3.64 mm), and trunk length from VP to the sacral point (3.37 mm). For the mannequin, the maximum lateral deviation of the spine to the left of VP-DM (2.90%) and the maximum surface rotation to the left (1.78%) had the greatest percent change between means. For human participants, pelvic tilt angle had the highest percent change (719.76%) followed by pelvic torsion (195.92%), rotation correction (123.16%), and pelvic tilt height difference (100.61%).

Within-scan variance for measurements on the mannequin was small (Table 5). For the mannequin, variance ranged from 0.0000 for rotation correction of the pelvis to 1.6175 for the kyphotic apex. For human participants, within-scan variance ranged from 0.05 for the angle of trunk inclination between VP-DM to 36.04 for the inflection point between the kyphotic apex and lordotic apex. A significant within-scan variance was found for each of the 40 spine shape parameters for human participants (all *P* < 0.001).

**Table 5:** Within-scan variance for the mannequin and human participants calculated using the standard average value (SAV)

Spine shape parameter by subgroup	Mannequin	Human participants
Localization and distance		
Trunk length VP-DM, mm	0.0006 (0.02)	2.79 (1.67)
Trunk length VP-SP, mm	0.0010 (0.03)	7.21 (2.69)
Trunk length VP-SP, %	0.0001 (0.01)	0.22 (0.47)
Dimple distance, mm	0.0006 (0.02)	3.22 (1.79)
Dimple distance, %	0.0001 (0.01)	0.15 (0.39)
Trunk and pelvis imbalances		
Trunk inclination VP-DM, °	0.0001 (0.01)	0.05 (0.22)
Trunk inclination VP-DM, mm	0.0005 (0.02)	3.69 (1.92)
Trunk imbalance VP-DM, °	0.0001 (0.01)	0.06 (0.24)
Trunk imbalance VP-DM, mm	0.0007 (0.03)	4.13 (2.03)
Pelvic tilt DL-DR, °	0.0006 (0.02)	0.90 (0.95)
Pelvic tilt DL-DR, mm	0.0010 (0.03)	2.28 (1.51)
Pelvic torsion DL-DR, °	0.0007 (0.03)	0.62 (0.79)
Pelvic inclination dimples, °	0.0190 (0.14)	1.01 (1.00)
Rotation correction pelvis, °	0.0000 (0.00)	0.35 (0.59)
Spinal reference points		
Inflection point ICT, mm	0.0043 (0.07)	6.10 (2.47)
Kyphotic apex, mm	1.6175 (1.27)	8.04 (2.84)
Inflection point ITL, mm	0.0321 (0.18)	36.04 (6.00)
Lordotic apex, mm	0.0320 (0.18)	10.72 (3.27)
Inflection point ILS, mm	0.0112 (0.11)	15.90 (3.99)
Fleche cervicale, mm	0.0011 (0.03)	2.32 (1.52)
Fleche lombaire, mm	0.0010 (0.03)	2.07 (1.44)
Fleche cervicale VP, mm	0.0011 (0.03)	2.88 (1.70)
Spinal curve measurements		
Kyphotic angle ICT-ITL, °	0.0007 (0.03)	1.02 (1.01)
Kyphotic angle VP-ITL, °	0.0006 (0.02)	1.54 (1.24)
Kyphotic angle VP-T12, °	0.0004 (0.02)	1.18 (1.09)
Lordotic angle ITL-ILS, °	0.0004 (0.02)	2.29 (1.51)
Lordotic angle ITL-DM, °	0.0002 (0.01)	1.92 (1.39)
Lordotic angle T12-DM, °	0.0005 (0.02)	1.75 (1.32)
Pelvic inclination, °	0.0094 (0.10)	0.73 (0.85)
Spinal deviation		
Surface rotation RMS, °	0.0003 (0.02)	0.29 (0.54)
Surface rotation max, °	0.0013 (0.04)	6.30 (2.51)
Surface rotation right, °	0.0013 (0.04)	1.36 (1.17)
Surface rotation left, °	0.0015 (0.04)	0.98 (0.99)
Surface rotation amplitude, °	0.0026 (0.05)	0.92 (0.96)
Trunk torsion, °	0.1244 (0.35)	2.66 (1.63)
Lateral deviation RMS, mm	0.0003 (0.02)	0.53 (0.73)
Lateral deviation, mm	0.0008 (0.03)	6.29 (2.51)
Lateral deviation right, mm	0.0008 (0.03)	1.79 (1.34)
Lateral deviation left, mm	0.0010 (0.03)	1.22 (1.10)
Lateral deviation amplitude, mm	0.0017 (0.04)	2.50 (1.58)

Data are reported as variance (within-scan SD). Variance for all human spine shape parameters was significant (*P* < 0.001)

*DL* left sacral dimple, *DM* middle point between the left and right sacral dimples, *DR* right sacral dimple, *ICT* cervical-thoracic inflection point, *ILS* lumbar-sacral inflection point, *ITL* thoracic-lumbar inflection point, *RMS* root mean square, *SP* sacral point, *VP* vertebral prominens

## Discussion

The current study was conducted to evaluate the difference between DRV and SAV for calculating spine shape parameters collected from the DIERS formetric 4D. Our results suggested that significant variability occurred within a scan and should be considered when evaluating the parameters of the DIERS formetric 4D. To optimally use the DIERS formetric 4D for longitudinal within-subject comparisons in research and clinical settings, investigators need to understand what the spine shape parameter values represent and the level of variability that occurs within a scan. This information will help researchers and clinicians to determine the level of change in spine shape parameters that can be attributed to an actual change rather than inherent variability that occurs within the human participant and instrument. To our knowledge, no previous studies have investigated the DIERS formetric 4D parameters with this level of critical within-scan analysis.

In the current study, completing scans on a mannequin allowed us to use a model of the human body to evaluate the instrument’s algorithms for DRV in comparison with the calculated SAV while eliminating the influence of postural sway and breathing on within-scan variability. The absolute difference between the mean DRV and mean SAV was very small for all of the spine shape parameters. The largest percent change observed between the mean DRV and mean SAV was for the maximum lateral deviation of VP-DM to the left, which had an absolute difference of only 0.03 mm. The extremely small within-scan variance observed throughout each of the spine shape parameters was an indication of the ability of the DIERS formetric 4D instrument to evaluate the static human shape with a high level of consistency. In this circumstance, the DRV provides an adequate estimation of the SAV.

Larger differences between the mean DRV and mean SAV for each parameter were observed for our human participants because of the added variability from postural sway and breathing. Nine of the 40 spine shape parameters had a large percent change (> 7%). Parameters in the localization and distance subgroup and the spinal curve measurements subgroup did not have a large percent change when comparing mean DRV and mean SAV.

The parameters with the largest percent change were from the trunk and pelvis imbalances subgroup. Six of the nine trunk and pelvis imbalances parameters had an extremely large percent change between the mean DRV and mean SAV: angle of trunk imbalance between VP-DM, trunk imbalance distance between VP-DM, pelvic tilt angle, pelvic tilt height difference, pelvic torsion, and rotation correction. The high percent change for these parameters may be attributed to their small mean DRV and mean SAV values. Parameters from the trunk and pelvis imbalances subgroup, such as pelvic tilt angle, pelvic tilt height difference, and pelvic torsion, have been reported as less reliable [7, 10] and more variable [8, 10]. Further, studies have attributed the increased variability and the decreased reliability of these parameters to outside influence from inconsistent patient positioning between scans [7, 8]. Although these studies [7, 8, 10] focused on between-scan variability and reliability, positioning seems to have influenced their results. In the current study, only a single scan was considered, and the participants remained in the same position during the entire scan, as recommended by the manufacturer. The mannequin we scanned lacked postural sway and breathing, and we found very low variability for pelvic tilt angle, pelvic tilt height difference, and pelvic torsion. This variability increased with the human participants even though the magnitude of the means were very small. Taken together, these results suggest that postural sway and breathing should be considered as a component of within-scan variability and are likely a meaningful contributor to the reported between-scan variability due to patient positioning [7, 8].

Previous studies support this approach. Schroeder et al. [10] suggested that measurement error may be influenced by individual variation in the soft tissue structure. Although BMI has not been found to influence the reliability of the DIERS formetric 4D in calculating spine shape parameters [9, 12], parameters related to the pelvis are calculated and represented based on the localization or a derivative of the localization of DL and DR. Since the location of DL and DR are correlated to but not necessarily representative of the underlying structures of the pelvis [13] and because the position of these landmarks is used to create the Cartesian coordinate plane for back shape reconstruction [14, 15], postural sway may manipulate the contour of the soft tissue that makes up either dimple. The change in soft tissue contour could influence the consistency at which the DIERS formetric 4D localizes DL and DR and increase variability in the evaluation of any parameter directly related to the pelvis. Although the current study did not investigate the effect of variability within the localization of DL and DR, clinicians and researchers should be aware that that observable changes within those landmarks may add to the within-scan variability and influence the ability to generate meaningful and comparable results.

Two of the nine spine shape parameters with a large percent change between the mean DRV and mean SAV were in the spinal deviation subgroup: trunk torsion and maximum surface rotation. The high percent change in trunk torsion is likely related to soft tissue changes. The maximum surface rotation evaluates the maximum rotation of the vertebra in either direction. Because this parameter accounts for the greatest rotation in either direction, a small change in posture because of normal postural sway during a scan has the potential to change the rotational characteristics of each vertebral segment and the location of the maximum rotation along the spine. Within the maximum surface rotation parameter, a change of direction or location could cause the value to flip from positive to negative, creating a large amount of variability. Therefore, the results of the current study show a larger percent change and variance for maximum surface rotation (percent change = 31.68%, variance = 6.30) than for maximum surface rotation to the right (percent change = 6.41%, variance = 1.36) and maximum surface rotation to the left (percent change = 3.85%, variance = 0.98). Without knowing the segment of maximum rotation, any observable change in maximum surface rotation which takes into account two directions along the entire length of the spine should be interpreted with caution.

One of the nine spine shape parameters with a large percent change between the mean DRV and mean SAV was in the spinal reference points subgroup: cervical-thoracic inflection point. To our knowledge, the reliability and variability of this spine shape parameter has not been previously reported. In the current study, a large increase in within-scan variance from the mannequin to the human participant was observed. Since the inflection point above the kyphotic apex is dependent on the change in surface curvature of the neck, any change in the head position because of postural sway or other reasons would be expected to influence the variability of this parameter. If the inflection point is a spine shape parameter of interest, because of the amount of mobility within the neck, more focus should be placed on finding a consistent stable position of the head.

Based on results of the current study and even though we observed large percent changes between DRV and SAV, the absolute differences between the mean DRV and mean SAV for each parameter in our human participants were small and likely not clinically meaningful. In addition, the inter-method reliability between DRV and SAV was excellent, indicating little difference between the two methods. Therefore, using either the DRV or the SAV is an acceptable method for evaluating spine shape parameters. The greatest determinant of which method to use may lie in the intention of the user. For instance, the DRV may currently be more useful for clinicians who want to quickly access parameter values, such as when looking at a pictorial representation of the posture. On the other hand, researchers may find the SAV is more meaningful when they want to observe the variability associated with each parameter. As found in the current study, when postural sway occurs, each of these parameters varies. Although no clinically relevant differences between DRV and SAV were observed, a significant within-scan variance was observed for all 40 of the human spine shape parameters, indicating that for an individual scan each parameter contained significant information not accounted for in the DRV. As such, we recommend that an indication of variability be reported to adequately represent the change that occurs from normal postural sway and breathing. Currently, to evaluate within-scan variability, data files from each image collected must be parsed, compiled, and analyzed. A representation of the within-scan variability inside the DIERS data collection and processing software would provide clinicians and researchers with immediate information to determine meaningful change whether they are evaluating the natural change of posture longitudinally or change from an intervention. Further, reporting variability will allow researchers to explore the normal range of changes that occur in the spine and pelvis during quiet stance and to determine normative ranges that can be used to understand meaningful change in future studies.

The current study had several limitations. One limitation is that the influence of head positioning on the spine shape parameters is unknown, but it is possible that variation in head placement may affect results. In anticipation of this limitation, a fixed point was provided as a visual reference in front of each participant near shoulder height in an attempt to establish a consistent head position. Future studies should investigate the effect of changing head positions on the spine shape parameters. Another limitation is that the landmarks (DL, DR, and VP) that the instrumentation automatically identifies may require repositioning. In nearly 45% of scans (399), at least 1 landmark had to be adjusted in 1 or more images because of improper localization by the DIERS system. The possibility of this adjustment is reported in the instrument’s operations manual, and the user is instructed to reposition the landmark to the correct location. Within our dataset of 900 scans, a total of 32,400 landmarks/data points could be adjusted. In nearly all of the 399 scans where marker location was adjusted, only 1–2 of the markers were adjusted for 1 of the 3 landmarks. So estimating for the entire dataset, only 798 of the 32,400 data points were adjusted, resulting in a conservative correction rate of 3%. While adjusting landmarks is not ideal and could be a cause of variability, such infrequently required modification better represents the true parameter value than if the landmark was left in its original position. Finally, the current study included participants with a BMI range from 25 to 35, which is a wider range than previously reported studies [9, 12]. We have no reason to believe our BMI range would influence our results. The variability and reliability for the wider BMI range are currently being investigated.

Current studies in progress are focusing on the influence of within-scan, within-day, between-day, and between-participant variability on the measured variability of the DIERS formetric 4D over time. Understanding these components will allow for the establishment of population-based normative spinal parameter ranges that could improve our understanding of what outcomes can be determined as meaningful change. Although previous studies [9, 12] have evaluated the influence of BMI on the reliability of the spine shape parameters, more definitive analysis of the influence of BMI and other measures, such as sex and body fat percent, should be evaluated as well. Future studies should also investigate how changes in the location of DL and DR influence the stability of parameters from postural sway or modifications in landmark localization by a technician during processing.

## Conclusions

In the current study, the minimal variability observed in the mannequin suggested the DIERS formetric 4D instrument had high within-scan reliability. The absolute difference between the mean DRV and mean SAV for human participants was small, and the inter-method reliability was excellent. Both the DRV and SAV provided comparable spine shape parameter values. Because significant within-scan variability was identified, reporting the SAV along with the within-scan variability will increase the clinical usefulness of each spine shape parameter, especially when researchers and clinicians are trying to determine when a clinically meaningful change has occurred.

## Abbreviations

BMI: body mass index
DL: left sacral dimple
DM: middle point between the left and right sacral dimples
DR: right sacral dimple
DRV: DIERS-reported value
ICC: intraclass correlation coefficient
SAV: standard average value
VP: vertebral prominens
